# Urine Hydrogen Peroxide Levels and Their Relation to Outcome in Patients with Sepsis, Septic Shock, and Major Burn Injury

**DOI:** 10.3390/biomedicines10040848

**Published:** 2022-04-05

**Authors:** Miklos Lipcsey, Maria Bergquist, Rebecca Sirén, Anders Larsson, Fredrik Huss, Jay Pravda, Mia Furebring, Jan Sjölin, Helena Janols

**Affiliations:** 1 Hedenstierna Laboratory, Department of Surgical Sciences, Uppsala University, 75185 Uppsala, Sweden; miklos.lipcsey@surgsci.uu.se; 2 Department of Medical Sciences, Clinical Physiology, Uppsala University, 75185 Uppsala, Sweden; 3 Department of Medicine, Danderyd Hospital, 18288 Stockholm, Sweden; rebecca.siren@hotmail.com; 4 Department of Medical Sciences, Clinical Chemistry, Uppsala University, 75185 Uppsala, Sweden; anders.larsson@akademiska.se; 5 Burn Center, Department of Plastic and Maxillofacial Surgery, Uppsala University Hospital, 75185 Uppsala, Sweden; fredrik.huss@akademiska.se; 6 Department of Surgical Sciences, Plastic Surgery, Uppsala University, 75185 Uppsala, Sweden; 7 Inflammatory Disease Research Centre, Therashock LLC, Palm Beach Gardens, FL 33410, USA; jaypravda@yahoo.com; 8 Department of Medical Sciences, Section of Infectious Diseases, Uppsala University, 75185 Uppsala, Sweden; mia.furebring@medsci.uu.se (M.F.); jan.sjolin@akademiska.se (J.S.); helena.janols@akademiska.se (H.J.)

**Keywords:** hydrogen peroxide, H_2_O_2_, sepsis, shock, burn injury, TBSA, mortality, AKI, hypermetabolism

## Abstract

Hydrogen peroxide (H_2_O_2_) and oxidative stress have been suggested as possible instigators of both the systemic inflammatory response and the increased vascular permeability associated with sepsis and septic shock. We measured H_2_O_2_ concentrations in the urine of 82 patients with severe infections, such as sepsis, septic shock, and infections not fulfilling sepsis-3 criteria, in patients with major burn injury with associated systemic inflammation, and healthy subjects. The mean concentrations of H_2_O_2_ were found to be lower in patients with severe infections compared to burn injury patients and healthy subjects. Patients with acute kidney injury (AKI), vs. those without AKI, in all diagnostic groups displayed higher concentrations of urine H_2_O_2_ (*p* < 0.001). Likewise, urine concentrations of H_2_O_2_ were higher in non-survivors as compared to survivors (*p* < 0.001) at day 28 in all diagnostic groups, as well as in patients with severe infections and burn injury (*p* < 0.001 for both). In this cohort, increased H_2_O_2_ in urine is thus associated with mortality in patients with sepsis and septic shock as well as in patients with burn injury.

## 1. Introduction

Sepsis is one of the most common causes of death in the intensive care unit despite advances in care [1]. Hydrogen peroxide (H_2_O_2_) and oxidative stress have been suggested to be possible instigators of both the systemic inflammatory response and the increased vascular permeability associated with sepsis and septic shock through their effects on vascular endothelial cells [2,3,4]. Physiological changes during sepsis generate a hypermetabolic state and an increase in the production of adenosine triphosphate (ATP) through the electron transport chain in mitochondria. This can lead to an oxidative burst, a rapid increase in released reactive oxygen species (ROS), superoxide (O_2_^−^), hydroxyl radicals (HO^−^), and H_2_O_2_. The patient’s prolonged state of increased metabolism has been much disputed as a possible reason for the deleterious effects of sepsis [2]. The possible microangiopathic effects of H_2_O_2_ can be summarized as systemic microcirculatory dysfunction through damage to distant capillary beds, microangiopathic oedema, and refractory hypotension [5,6]; all distinguishing characteristics of septic shock.

A study investigating ROS production in patients with septic shock showed that plasma from these patients induces ROS formation in naïve HUVECs (Human Umbilical Vein Endothelial Cell) [6]. They also found that the elevated ROS-induction rate in HUVECs correlated with mortality rates, SAPS II (Simplified Acute Physiology Score), and SOFA scores (Sequential Organ Failure Assessment) in individuals with septic shock. These findings strengthen the hypothesis that ROS and H_2_O_2_ may be of key importance for the pathophysiology of sepsis and septic shock. Toxic levels of H_2_O_2_ have been shown to result in both laboratory and clinical abnormalities associated with sepsis, such as immunosuppression, bioenergetic organ failure, and hypotension [2,7].

Major burn injury results in a similar systemic inflammatory response, triggering multi-organ dysfunction and disturbances in metabolism that can last for 24 months or longer, after the time of injury [8,9,10]. Oxidative stress is one of the main contributors to the negative pathophysiological changes associated with traumatic burn injury, which is brought on by increased neutrophil activity and elevated xanthine oxidase activity. In turn, this process produces toxic byproducts such as H_2_O_2_ and its conversion product, the hydroxyl radical, causing damage to dermal vascular endothelial cells and leading to increased vascular permeability [11,12,13].

Despite studies performed on H_2_O_2_ in systemic inflammation, there is still a lack of knowledge of its role in sepsis and burn injury. Consequently, we conducted a prospective observational study in patients with severe infections, patients with burn injuries—i.e., systemic inflammation—and healthy controls measuring H_2_O_2_ in urine. As H_2_O_2_ is considered to be somewhat more stable in urine than in plasma, and as we specifically wanted to explore H_2_O_2_ concentrations in patients with and without acute kidney injury (AKI), we focused our analysis on urine samples from these patient groups.

The aim of the present study was to quantify H_2_O_2_ in the urine of patients with major burn injuries, representing non-infectious severe inflammation, and to compare the concentrations with those of patients with sepsis and septic shock on day 1, 2, 3, and 5–6 after hospital admission. Furthermore, we wanted to explore if H_2_O_2_ concentration was associated with 28-day mortality, organ failure, and common inflammatory mediators, such as C-reactive protein (CRP) and procalcitonin.

## 2. Materials and Methods

### 2.1. Setting

This study was designed as a prospective descriptive study to investigate H_2_O_2_ concentrations in patients with burn injury, sepsis, or septic shock. Healthy subjects were also recruited to determine reference values of H_2_O_2_. The study was conducted at Uppsala University Hospital, Sweden. STROBE guidelines were followed for reporting.

### 2.2. Ethical Approval

Permission for the study was granted by The Regional Ethical Review Board in Uppsala (Dnr 2015/081). Participating patients and healthy volunteers were supplied with information about the study and gave written informed consent for participation. If a participant was deemed incapable of providing consent at the time of initiation, written consent was first obtained from the patient’s next of kin and later from the participant. The Helsinki declaration and its subsequent revisions were followed.

### 2.3. Patient Groups

Patients were included in the study from the infectious disease wards and the general intensive care units, while patients with major burn injuries were included from new admissions at the burn intensive care unit between the 1st of November 2015 and the 31st of May 2018. Healthy subjects were recruited among consenting hospital employees to determine concentrations of circulating H_2_O_2_ in a healthy population. The inclusion criteria for the patient groups were adults (≥18 years) and diagnosed within 24 h of arrival to the hospital with any of the following: (1) Major burn injury (for this study defined as >10% full-thickness burn injury), (2) Severe sepsis, or (3) Septic shock, adhering to the 2001 Sepsis-2 definition. Burn patients were required to display >2 of the following clinical SIRS criteria: Temperature > 38 °C or <36 °C; Heart rate > 90 beats/min; Respiratory rate > 20 breaths/minute or PaCO_2_ < 4.3 kPa; WBC > 12 × 10^9^/L or <4 × 10^9^/L, or >10% immature neutrophils [14]. Exclusion criteria were age < 18 years, pregnancy, and patients unlikely to survive >48 h due to any other underlying disease than sepsis. In the protocol, sepsis was defined as an infection in combination with >2 SIRS criteria. Accordingly, severe sepsis was defined as sepsis complicated by sepsis-induced hypotension, hypoperfusion, or organ dysfunction. Septic shock was defined as sepsis with manifest hypotension resistant to adequate fluid resuscitation and hypoperfusion or organ dysfunction [15]. Sepsis is currently defined according to the Sepsis-3 criteria [16] with suspected or verified infection and an increase of two or more in SOFA score. Septic shock is defined in Sepsis-3 as sepsis and serum lactate over 2 mmol/L and vasopressor treatment. The Sepsis-3 definition was used for the main analysis.

### 2.4. Data Collection

Demographics, daily urine output, fluids, and any blood transfusions were registered, as well as clinical variables relevant for the degree of inflammation and organ dysfunction (plasma creatinine, plasma bilirubin, maximal daily vasoactive medication dose, ratio of arterial oxygen partial pressure to fractional inspired oxygen, PaO_2_/FiO_2_) for each ICU day where applicable. Medications of interest, such as catecholamines and steroids, and any radiological or microbial findings of importance for patient diagnosis were documented. Blood gas values and clinical laboratory data (CRP, leucocyte and platelet counts (PLT), and microbiological cultures) were obtained from laboratory records. Arterial blood gas data were collected according to clinical routines but were at a minimum collected at admission and then daily in non-ICU patients. If arterial blood gas was unattainable, a venous blood gas and saturation (pulse oximetry) was registered instead. A Sequential Organ Failure Assessment (SOFA) score was calculated for the admission day. Acute kidney injury was defined according to the KDIGO criteria [17]. Mortality was registered at 28 days post-hospital admission. As samples from healthy subjects were only collected at one time point, laboratory analysis of these samples was limited to plasma and urine analysis for H_2_O_2_.

### 2.5. Sample Collection

Study-specific blood samples were collected from patients on days 1, 2, 3, and 5–6. Urine samples were collected either using a syringe through the membrane of the urinary catheter or by utilizing a urine vacutainer straw. The EDTA plasma and urine samples were sent to the laboratory immediately, processed, and then stored at −80 °C until analysis.

### 2.6. Sample Analyses

All routine blood tests were analyzed at the hospital’s routine laboratory of Clinical Chemistry and Pharmacology, Uppsala University Hospital. Routine laboratory analysis included: high sensitivity C-reactive protein (CRP), procalcitonin, a complete blood count (CBC), and electrolytes including sodium, potassium, and creatinine. The analysis of hydrogen peroxide (plasma and urine) was performed using Arbor Assays™: DetectX^®®^ Hydrogen Peroxide Colorimetric Activity Kit (Arbor Assays, Ann Arbor, MI, USA) according to the manufacturer’s instructions by one technician. The limit of detection was determined as 1.96 µM and the coefficient of variation was approximately 5%.

Plasma IL-6 and neutrophil gelatinase-associated lipocalin (NGAL) were analyzed using the Human IL-6 and NGAL Duoset ELISA kits (DY206 and DY1757, R&D Systems, Minneapolis, MN, USA) according to the manufacturer’s instructions. All ELISA data were acquired using the SpectraMax^®®^ plate reader (Fisher Scientific GTF AB, Gothenburg, Sweden) with its supplied functions and software.

### 2.7. Statistical Analysis

Given the pilot nature of this study, no power analysis was performed. Data were assessed for normality. Data with logarithmic distribution were log-transformed. Data are presented as a median (inter-quartile range, IQR) or a number of observations (percent of the total number of observations). The associations between two variables were assessed with a Spearman’s rank-order correlation test. The differences between the patient groups on day one were tested using Kruskal–Wallis with Dunn’s correction for multiple comparisons. The changes over time for normally distributed variables were assessed by ANOVA type III for repeated measures. Odds ratios for AKI and death at 28 days were calculated in a univariate logistic regression model. As the inclusion criteria were according to Sepsis-2 [15] definitions but data were analyzed according to the Sepsis-3 definitions [16], we performed a sensitivity analysis with Sepsis-2 definitions for the main outcome. STATISTICA version 13.2 (StatSoft, Tulsa, OK, USA) and GraphPad Prism 9.0 for Windows (GraphPad Software Inc., La Jolla, CA, USA) were used for the calculations and figures. A *p* < 0.05 was considered statistically significant where relevant. Given the explorative nature of this study, multiple comparisons were not corrected for.

## 3. Results

The patients were enrolled continuously over a period of approximately 2.5 years. In total, 82 patients and 38 healthy subjects were included in the study. The demographics of patients in the different diagnosis groups are presented in Table 1. The microbiological findings in the cohort are presented in Supplementary Table S1. Among the patients with burn injury, representing severe inflammation without infection, no patients had positive blood cultures or a clinically established secondary infection during the study. The mortality on day 28 was higher in the group of patients with burn injury compared to the other diagnosis groups (*p* = 0.01).

Patients with septic shock displayed higher levels of plasma CRP on day 1 compared to the other patients (*p* < 0.001). The patients with burn injury displayed peak concentrations of plasma CRP and NGAL on days 5–6. On day 1, plasma creatinine (*p* = 0.02), IL-6 (*p* = 0.002), and NGAL (*p* < 0.0001) were highest in the septic shock patients (Supplementary Table S2). Urine (*n* = 23) and plasma (*n* = 25) samples were collected from healthy subjects with a median age of 61 years (47–64 IQR) and displayed H_2_O_2_ concentrations of 9.8 (6.7–14.6) µM in the urine. No difference was observed between male or female patients in concentrations of H_2_O_2_ in urine (*p* = 0.96). Healthy subjects had higher urine H_2_O_2_ than patients with sepsis and septic shock (*p* < 0.05 for both).

### 3.1. H_2_O_2_ Concentrations in Critically Ill Patients

**Figure 1:**
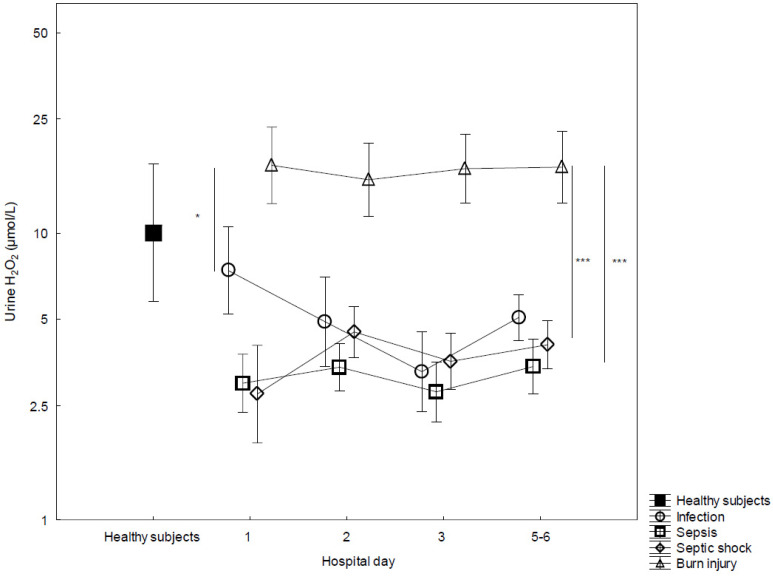
Concentrations of urine H_2_O_2_ from admission (day 1) to day 5–6 in the ICU, stratified by sepsis (*n* = 37), septic shock (*n* = 23), infection (*n* = 4) and major burn injury (*n* = 18). H_2_O_2_ concentrations in urine from healthy subjects (*n* = 23) at one timepoint. ANOVA III for repeated measures was used to assess differences over time. Mean ± standard error of the mean (SEM). * *p* < 0.05, *** *p* < 0.001.

We measured H_2_O_2_ concentrations in urine from 82 patients with major burn injury with associated (non-infectious) systemic inflammation, sepsis, septic shock, and patients with infection not fulfilling Sepsis-3 criteria. The concentrations of H_2_O_2_ in urine from the patient groups are illustrated in Figure 1. The highest mean concentrations of urine H_2_O_2_ were found in patients with burn injury. We did not observe any changes over time during the first six days following ICU admission in any of the diagnosis groups. The H_2_O_2_ concentrations observed in urine from burn injury patients were not statistically different to those found in healthy subjects.

There was no difference in urine H_2_O_2_ in patients with or without steroid treatment after adjusting for the diagnostic group. Patients with positive blood cultures displayed higher concentrations of H_2_O_2_ in urine compared to patients with negative blood cultures (*p* < 0.001).

In the sensitivity analysis using Sepsis-2 criteria, urine H_2_O_2_ was higher in patients with burn injury compared to patients with severe sepsis and septic shock (*p* < 0.001). There was no difference in urine H_2_O_2_ between patients with sepsis and septic shock.

The H_2_O_2_ concentrations were also analyzed in plasma from the patients and healthy subjects (Figures S1–S3). The plasma from healthy subjects displayed H_2_O_2_ concentrations of 10.4 (7.6–15.2) µM. The results in plasma correspond to the data in urine, however, as the H_2_O_2_ assay used is not validated by the manufacturer for analysis in plasma, we base our conclusions solely on the data from the urine.

### 3.2. H_2_O_2_ Association with Outcome

**Figure 2:**
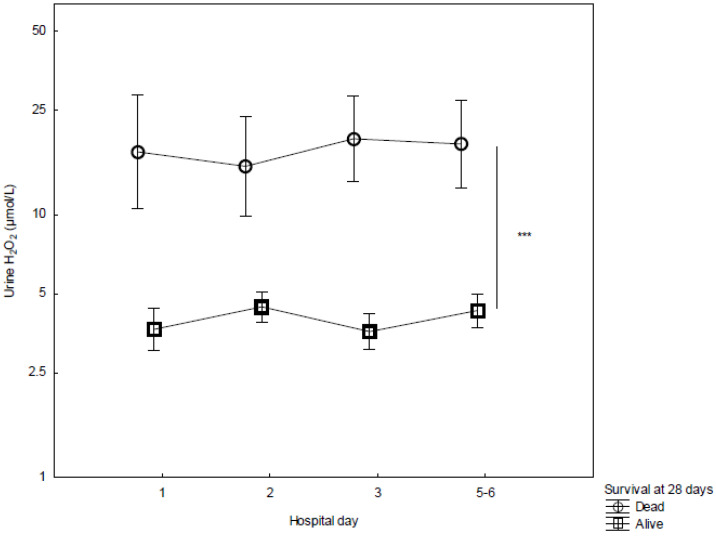
Concentrations of urine H_2_O_2_ from admission (day 1) to day 5–6 in the ICU, stratified by survivors (*n* = 71) and non-survivors (*n* = 11). ANOVA III for repeated measures was used to assess differences over time. Mean ± SEM. *** *p* < 0.001.

**Figure 3:**
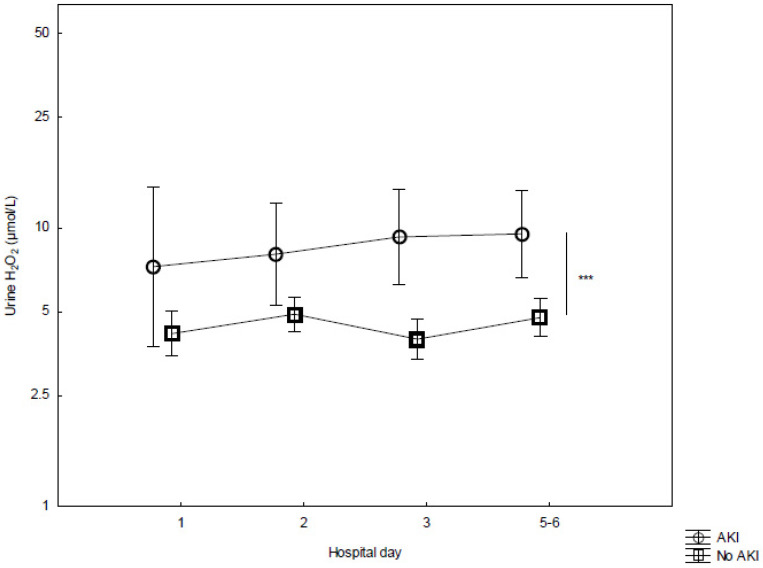
Concentrations of urine H_2_O_2_ from admission (day 1) to day 5–6 in the ICU, stratified by patients with acute kidney injury (AKI) (*n* = 12) and without AKI (*n* = 70). ANOVA III for repeated measures was used to assess differences over time. Mean ± SEM. *** *p* < 0.001.

**Figure 4:**
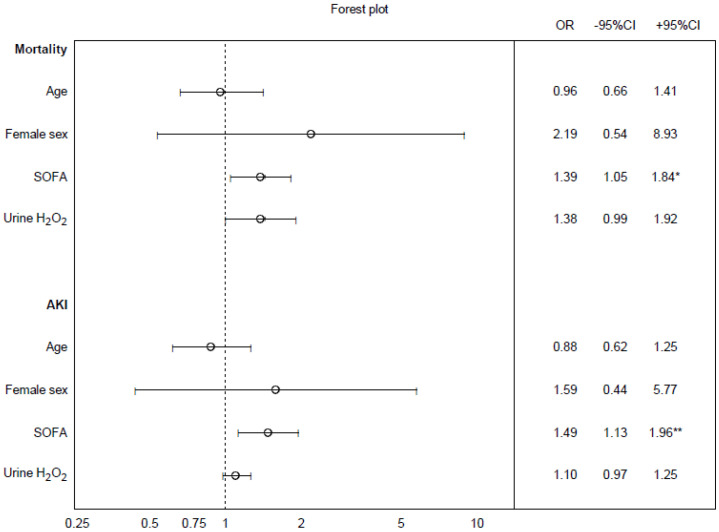
Forest plot. Odds ratios for univariable logistic regression. Age has the unit of decades and H_2_O_2_ has the unit 10 µmol/L to optimize presentation in the figure. Odds ratio ± 95% confidence interval ( CI). * *p* < 0.05, ** *p* < 0.01.

Urine concentrations of H_2_O_2_ were higher in non-survivors as compared to survivors in the whole cohort (*p* < 0.001, Figure 2). This difference could be observed already upon admission and was maintained over the study period. The patients with AKI displayed higher concentrations of urine H_2_O_2_ than those without AKI (*p* < 0.001, Figure 3). Apart from the SOFA score on day 1, neither age nor sex predicted AKI or 28-day mortality (Figure 4). The observed difference in the urine concentrations of H_2_O_2_ were also present between non-survivors as compared to the survivors in the group of patients with infection, sepsis, and septic shock, as well as the burn injury patients (Figure S4). However, there is no statistically significant difference between the patients with and without AKI in any of these patient groups alone (Figure S5).

### 3.3. H_2_O_2_ Correlations to Clinical Parameters

The correlation heatmap of the extreme levels of biomarkers is presented in Figure S6. Neither age (rho = −0.09) nor SOFA (rho = 0.12) correlated with the H_2_O_2_ concentrations in urine on admission or on any of the hospital days (day 1 rho = 0.05, day 2 = 0.11, day 3 = 0.21, day 5–6 = −0.08). PCT (rho = −0.34, *p* = 0.003) and NGAL (rho = −0.27, *p* = 0.02) displayed negative correlations with the H_2_O_2_-concentrations in the urine on admission, while PaO_2_ (rho = 0.23, *p* = 0.04) displayed a positive correlation with the H_2_O_2_-concentrations in the urine during the same time point.

## 4. Discussion

In this prospective observational study in patients with severe infections (sepsis, septic shock, and infection without sepsis/septic shock) and major burn injuries (non-infectious inflammation), the urine concentrations of H_2_O_2_ were lower in patients with severe infections than in those with burn injuries and healthy controls. Patients with positive blood cultures, AKI, and patients who did not survive to day 28 after admission had higher urine concentrations of H_2_O_2_.

We did not observe a difference in the concentrations of urine H_2_O_2_ between the whole cohort and the healthy subjects included in this study, which is in line with previous results of urine H_2_O_2_ in sepsis patients compared to healthy controls [18]. In human plasma, a normal range for H_2_O_2_ has been demonstrated to be around 1–5 μM [19,20] and concentrations as low as 10 μM have been suggested to be stressful for cells [19]. However, in freshly voided human urine, significantly higher concentrations of H_2_O_2_ have been found, sometimes exceeding 100 μM [21]. This may be explained by the fact that H_2_O_2_ in urine can be increased by exercise [22], diet [21], and coffee [23], for example. Thus, the more relevant comparison of urine H_2_O_2_ is between different patient groups with severe inflammation rather than the comparison to healthy subjects.

Cumulative evidence suggests that H_2_O_2_ is playing a role in the development of sepsis and associated multi-organ failure [2,7]. Under normal conditions, H_2_O_2_, which is produced as a byproduct of the electron transport chain would be eliminated. However, in the case of severe systemic inflammation and the consequent hypermetabolism and oxidative burst, H_2_O_2_ can accumulate in cells and tissues to injurious levels with subsequent toxic exposure to this potent oxidizing metabolite [21]. The extent of H_2_O_2_ flux from the mitochondria to the plasma is not known, but H_2_O_2_ is potentially contributing to the lymphopenia commonly seen in sepsis [24,25], COVID-19 [26], and following burn injury [27]. It may, however, be proposed that the level of actual inflammation affects the production of H_2_O_2_. Interestingly, a study comparing patients with sepsis to healthy controls found that plasma superoxide dismutase (SOD) and catalase activity (CAT) were attenuated in patients with sepsis, and that these enzyme activities were inversely correlated to SOFA scores and inflammatory cytokines [28]. As SOD catalyzes the conversion of O_2_- into H_2_O_2_, a decreased activity of this antioxidant enzyme is likely to lead to decreased levels of H_2_O_2_ [29].

In this study, we observed higher urine H_2_O_2_-concentrations in patients who died in the first 28 days following ICU admission compared to patients who survived. This finding was consistent in the subgroups of patients with severe infections and burn injuries and suggests that H_2_O_2_ is associated with a poor prognosis in these patients. In line with this, a previous study in patients with sepsis and ARDS has demonstrated higher concentrations of H_2_O_2_ in the urine of patients who died compared to patients who survived [18]. However, in our study, it is possible that the degree of morbidity affects the H_2_O_2_ production and drives the mortality signal. The dynamics between groups regarding inflammatory parameters (CRP, PCT, and WBC) appear to be dependent on elapsed time from the inflammatory onset. We found that patients with sepsis and septic shock had the highest CRP levels on day 2, whereas patients with burn injury presented with the highest CRP concentrations by day 5–6. This suggests that the immune response in patients with sepsis may have been initiated in the days before ICU admission, relative to patients with burn injury who are usually admitted to care within the first 24 h after the injury. We can therefore speculate that if the increase in H_2_O_2_ is transient in severe infection, H_2_O_2_ may have decreased by the time patients are admitted to intensive care. This may also explain why we did not find strong correlations between the peak values of H_2_O_2_ and biomarkers of inflammation and organ function. NGAL levels peaked on day 1 in patients with sepsis and septic shock but reached their highest levels in patients with burn injury by day 5–6. Creatinine levels also peaked on day 1 in patients with sepsis and septic shock but reached their highest levels in patients with burn injury one day later, on day 2. Elevated NGAL and creatinine levels indicate increased strain on the kidneys during the initial phase of septic shock [30]. In this patient population, although plasma creatinine did not correlate with urine H_2_O_2_-concentrations, patients who developed AKI presented with higher concentrations of urine H_2_O_2_-concentrations. A previous study demonstrated increased plasma H_2_O_2_-concentrations in patients with sepsis-associated AKI [31]. The same study demonstrated the protective effects of exogenous H_2_S in an LPS-induced mouse model of AKI through the inhibition of inflammation and oxidative stress. If this mechanism could be confirmed in patients, it would support the hypothesis of the toxic effects of H_2_O_2_, as H_2_S is able to upregulate antioxidant response elements to produce the efficient H_2_O_2_ scavenger glutathione persulfide (GSSH) [32].

### 4.1. Strengths and Limitations

As far as we know, this is the first study that reports urine H_2_O_2_-concentration levels in patients with severe infections and major burn injuries. The study is prospective and the urine H_2_O_2_-concentrations were measured repeatedly for six days after admission. A further asset of the study is that patients with different severity of illnesses were included, as well as healthy subjects. To strengthen our observed data in urine, we performed the same analysis in plasma samples. While H_2_O_2_-concentrations in plasma harmonize with urine data, the assay is not intended for human plasma samples, which is why we have not based our conclusions on the observations found in plasma.

Our study has several limitations. First, as this was a pilot study, no power analysis was performed in advance to estimate the sample size. However, the data presented here is valuable for our understanding of H_2_O_2_ in these patients and may inform sample size considerations for future studies. Second, a limitation of this study is that patients with major burn injury had higher severity of illness and mortality than those with severe infections, as we wanted to compare urine H_2_O_2_-concentration levels in patients with infectious and non-infectious systemic inflammation. Third, the levels of antioxidants were not measured. Depletion of antioxidants may have occurred after the sustained increase in H_2_O_2_, and the concentrations of H_2_O_2_ observed in patients with sepsis and septic shock in this study could be reflective of pro- and anti-inflammatory mechanisms as well as the production of glutathione. Moreover, the urine H_2_O_2_-concentrations were measured in spot samples that may not reflect changes in concentrations over 24 h. However, a spot sampling was chosen instead of a 24-h urine collection to avoid the degradation of H_2_O_2_. Finally, the study cohort was originally designed to be stratified according to the Sepsis-2 criteria [15], but given the widespread use of Sepsis-3 criteria by the time data acquisition was done, we chose to analyze data according to the Sepsis-3 criteria [16]. However, a sensitivity analysis showed that the conclusions on the urine H_2_O_2_ levels would not change depending on which sepsis criteria were used.

### 4.2. Clinical Relevance

Although higher H_2_O_2_ concentrations in this cohort were associated with less favorable outcomes in terms of morbidity and mortality, our data did not provide sufficient guidance on the use of therapeutic concepts to decrease H_2_O_2_ levels in sepsis patients. However, this could be further investigated for patients with burn injuries.

### 4.3. Future Studies

Our data show that urine H_2_O_2_ concentrations were associated with AKI and mortality in patients with severe infections and major burn injuries, and it would add value for future studies to confirm this finding in blood from these patients. Since antioxidants were not quantified in this study, more studies are needed to explore their concentrations in these patients and their interplay with H_2_O_2_. Also, elevated urine H_2_O_2_ concentrations were seen in patients with AKI in this cohort, suggesting that the causality between these should be investigated. Quantifying the H_2_O_2_ and antioxidant concentrations in tissue or blister fluid may provide more information about its mechanism after burn injury. In the current study, we did not observe any changes over time in any of the diagnosis groups between days 1 to 6 following hospital admission. Given that the concentrations of H_2_O_2_ have been demonstrated to change within 1–2 h in urine [23], future studies could explore potential changes in H_2_O_2_ in these patient groups with a higher temporal resolution.

## 5. Conclusions

In this cohort, the urine concentrations of H_2_O_2_ were lower in patients with sepsis and septic shock than in those with major burn injuries and healthy controls during the first days following admission, and higher H_2_O_2_ concentrations were associated with mortality at 28 days.

## Figures and Tables

**Table 1:** Patient demographics and clinical parameters. Data are presented as a median (IQR) unless otherwise stated.

	All Patients (*n* = 82)	Septic Shock (*n* = 23, 28%)	Sepsis (*n* = 37, 45%)	Infection (*n* = 4, 5%)	Burn Injury (*n* = 18, 22%)
Age, years	70 (60–74)	72 (66–79)	70 (64–75)	62 (54–69)	67 (38–71)
Male sex, *n* (%)	35 (43)	9 (39)	19 (51)	2 (50)	6 (33)
SOFA ICU day 1	6 (4–8)	7 (5–9)	5 (4–7)	1 (1–1)	6 (4–9)
Continuous renal replacement therapy *n* (%)	3 (4)	1 (4)	0	0	2 (11)
Burned TBSA (%)					40 (27–54)
Full thickness burn (%)					33 (15–44)
Mortality at 28 days, *n* (%)	11 (13)	4 (17)	1 (3)	0	6 (33)
AKI, *n* (%)	12 (15)	5 (22)	2 (5)	0	5 (28)
Total urine/24 h (mL)	1065 (450–1825)	1000 (300–1610)	1090 (530–1825)	1160 (773–1585)	1240 (575–1905)
Received propofol (%)	25 (30)	5 (22)	2 (5)	0	18 (100)
Received Steroid treatment (%)	24 (29)	10 (43)	11 (30)	2 (50)	1 (6)
Positive blood culture	38 (46)	18 (78)	20 (54)	0	0 (0)
Infection foci					
Respiratory	21 (26)	7 (30)	14 (38)	0	0
Intra-Abdominal	5 (6)	4 (17)	1 (3)	0	0
Urogenital	24 (29)	9 (39)	14 (38)	1 (25)	0
Skin/Soft tissue	7 (9)	2 (9)	3 (8)	2 (50)	0
Unknown focus	6 (7)	1 (4)	4 (11)	1 (25)	0

AKI: acute kidney injury, ICU: Intensive care unit SOFA: sequential organ failure assessment, TBSA: Total Body Surface Area.

## Data Availability

The datasets used and/or analyzed during the current study are available from the corresponding author on reasonable request.

## Supplementary Materials

The following supporting information can be downloaded at: https://www.mdpi.com/article/10.3390/biomedicines10040848/s1, Figure S1. Concentrations of plasma H_2_O_2_ from admission (Day 1) to Day 5–6 in the ICU, stratified by sepsis (*n* = 37), septic shock (*n* = 23), infection (*n* = 4) and major burn injury (*n* = 18); Figure S2. Concentrations of plasma H_2_O_2_ from admission (Day 1) to Day 5–6 in the ICU, stratified by survivors (*n* = 71) and non-survivors (*n* = 11); Figure S3. Concentrations of plasma H_2_O_2_ from admission (Day 1) to Day 5–6 in the ICU, stratified by patients with acute kidney injury (AKI) (*n* = 12) and without AKI (*n* = 70); Figure S4. Concentrations of urine H_2_O_2_ from admission (Day 1) to Day 5–6 in the ICU, stratified by survivors and non-survivors in (A.) patients with infection, sepsis and septic shock and in (B.) patients with major burn injuries; Figure S5. Concentrations of urine H_2_O_2_ from admission (Day 1) to Day 5–6 in the ICU, stratified by patients with acute kidney injury (AKI) and without AKI in (A.) patients with infection, sepsis and septic shock and in (B.) patients with major burn injuries; Figure S6. Heatmap showing associations between urine H_2_O_2_ and variables of organ failure, inflammation and hypoperfusion assessed with Spearman’s rank-order correlation test; Table S1. Clinically significant microbiological findings in blood, urine, nasopharynx or the site of infection; Table S2. Clinical parameters. Data are presented as median (IQR) unless otherwise stated.
