# Supplementary material for: Urine Hydrogen Peroxide Levels and Their Relation to Outcome in Patients with Sepsis, Septic Shock, and Major Burn Injury
Source: Biomedicines. 2022 Apr 5;10(4):848. doi: 10.3390/biomedicines10040848 (PMC9030456; doi:10.3390/biomedicines10040848)
Supplement: Supplementary file 1 [file biomedicines-10-00848-s001.zip › biomedicines-1673410-supplementary.pdf]

biomedicines

MDPI

Supplementary materials

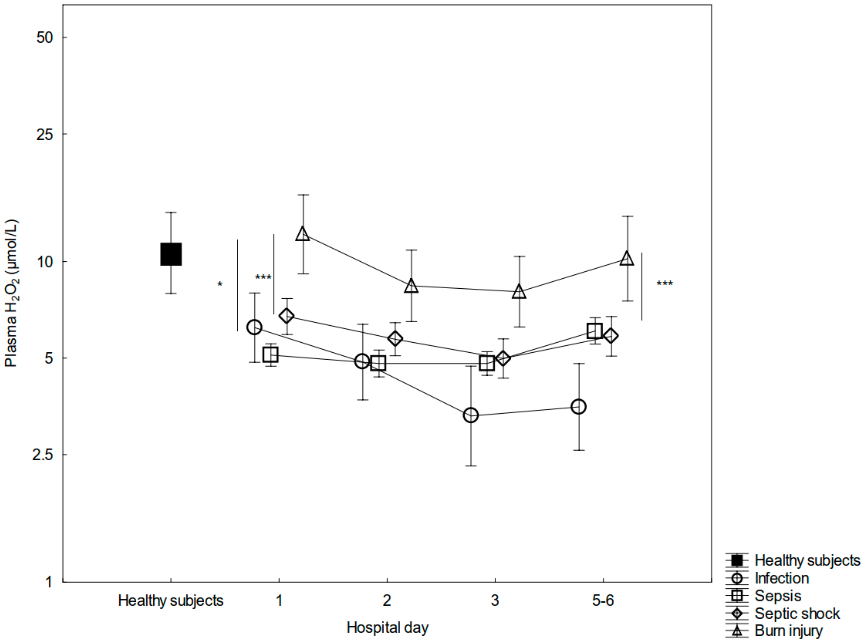

Figure S1. Concentrations of plasma H_2O_2 from admission (Day 1) to Day 5-6 in the ICU, stratified by sepsis (*n* = 37), septic shock (*n* = 23), infection (*n* = 4) and major burn injury (*n* = 18). H_2O_2 concentrations in plasma from healthy subjects (*n* = 23) at one timepoint. ANOVA III for repeated measures was used to assess differences over time. Mean ± SEM. *p <0.05, ***p <0.001.

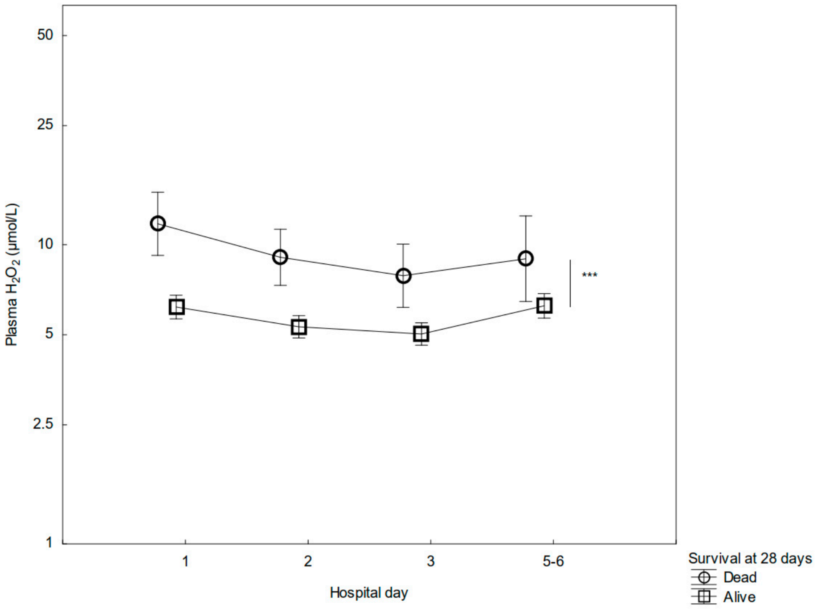

Figure S2. Concentrations of plasma H_2O_2 from admission (Day 1) to Day 5-6 in the ICU, stratified by survivors (*n* = 71) and non-survivors (*n* = 11). ANOVA III for repeated

measures was used to assess differences over time. Mean ± SEM. ***p <0.001

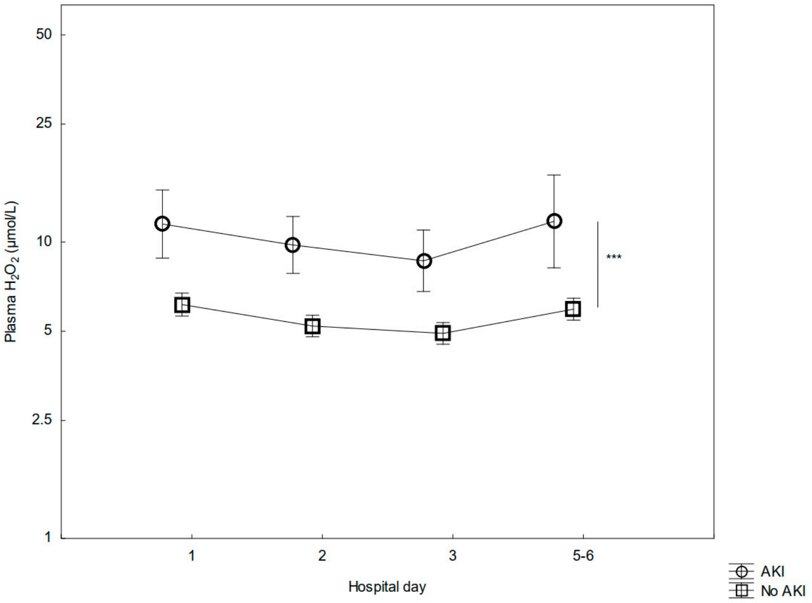

Figure S3. Concentrations of plasma H_2O_2 from admission (Day 1) to Day 5-6 in the ICU, stratified by patients with acute kidney injury (AKI) (n = 12) and without AKI (n = 70). ANOVA III for repeated measures was used to assess differences over time. Mean ± SEM. *** p <0.001.

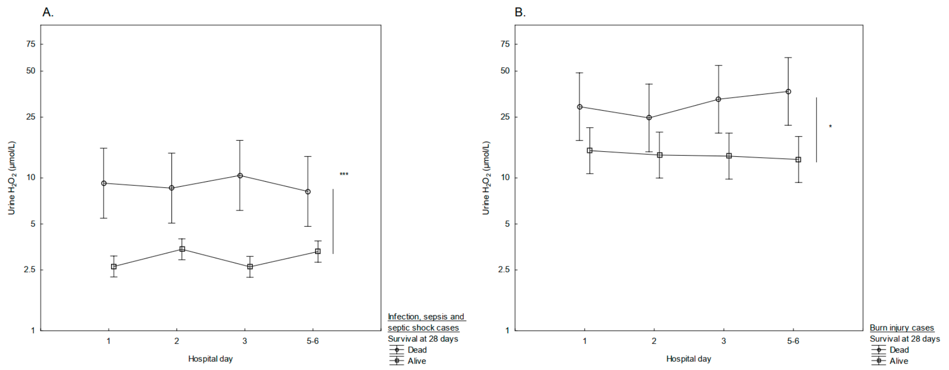

Figure S4. Concentrations of urine H_2O_2 from admission (Day 1) to Day 5-6 in the ICU, stratified by survivors and non-survivors in (**A**.) patients with infection, sepsis and septic shock and in (**B**.) patients with major burn injuries. ANOVA III for repeated measures was used to assess differences over time. Mean ± SEM. *p < 0.05, ***p <0.001

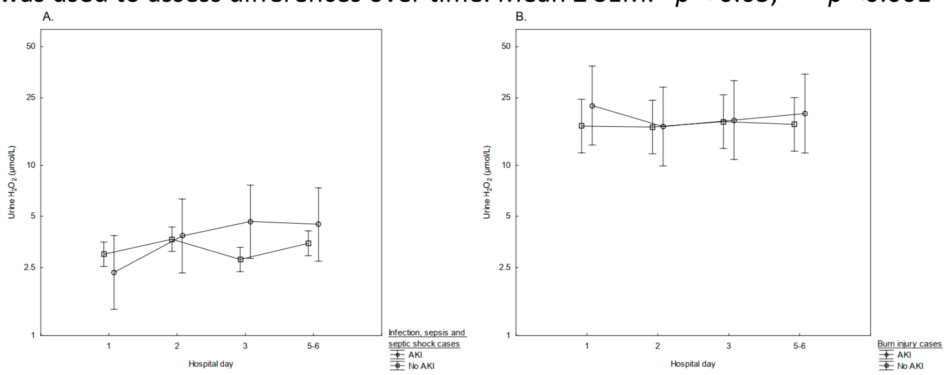

Figure S5. Concentrations of urine H_2O_2 from admission (Day 1) to Day 5-6 in the ICU, stratified by patients with acute kidney injury (AKI) and without AKI in (**A**.) patients with infection, sepsis and septic shock and in (**B**.) patients with major burn injuries. ANOVA III for repeated measures was used to assess differences over time. Mean ± SEM. *** $p < 0.001$.

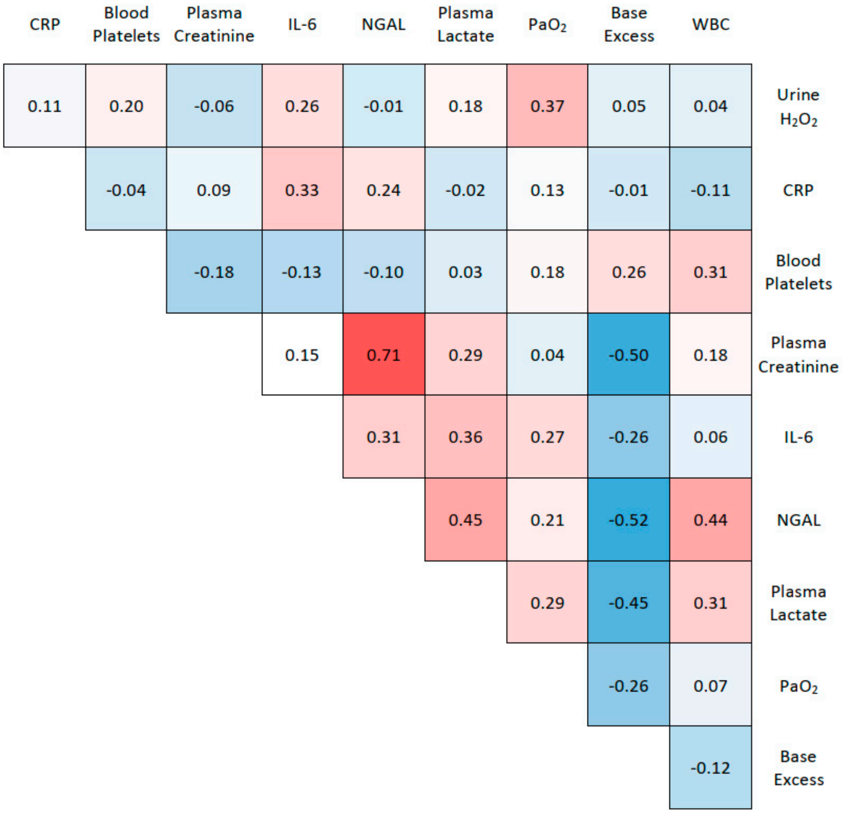

Figure S6. Heatmap showing associations between urine H_2O_2 and variables of organ failure, inflammation and hypoperfusion assessed with Spearman's rank-order correlation test. Correlations coefficients are presented as rho. CRP: C-reactive protein, IL-6: Interleukin 6, NGAL: neutrophil gelatinase-associated lipocalin, PaO_2:Arterial oxygen tension

Table S1. Clinically significant microbiological findings in blood, urine, nasopharynx or the site of infection.

	Number of cultures	% of the cohort
S. Aureus	5	6
Streptococcus species	15	18
S. Pneumoniae	10	12
S. Viridans	1	1
S. anginosus	1	1
Group A Streptococcus	1	1
Group G Streptococci	2	2

E. coli	17	21
E. coli (ESBL)	2	2
H. influenzae	2	2
K. pneumoniae	1	1
K. variicola	1	1
M. catarrhalis	1	1
P. aeruginosa	1	1
P. mirabilis	2	2
Negative cultures	35	43

Table S2. Clinical parameters. Data are presented as median (IQR) unless otherwise stated

	ALL PATIENTS (N=82)	SEPTIC SHOCK (*N* = 23, 28%)	SEPSIS (N = 37, 45%)	INFECTION (N = 4, 5%)	BURN INJURY (N = 18, 22%)
**PLASMA CRP [MG/L]**					
DAY 1	167 (87-253)	216 (152-279)	229 (152-314)	55 (48-109)	19 (8-93)
DAY 2	235 (158-306)	300 (232-316)	239 (205-304)	222 (164-265)	100 (58-176)
DAY 3	193 (145-286)	254 (158-299)	194 (144-249)	172 (146-187)	175 (123-294)
DAY 5-6	105 (60-205)	96 (66-116)	84 (40-155)	93 (73-123)	264 (152-314)
**PLASMA PCT [µG/L]**					
DAY 1	17 (2-60)	60 (34-98)	17 (3-51)	2 (2-8)	2 (0-4)
**BLOOD PLT [$X10^{12}$]**					
DAY 1	166 (98-236)	109 (78-180)	159 (101-215)	210 (182-272)	250 (147-385)
DAY 2	130 (76-189)	95 (57-148)	143 (81-187)	182 (133-245)	123 (85-233)
DAY 3	120 (68-183)	91 (57-144)	141 (89-194)	183 (143-228)	106 (59-158)
DAY 5-6	153 (79-228)	112 (69-203)	199 (122-239)	192 (157-246)	107 (67-160)
**PLASMA CREATININE [µMOL/L]**					
DAY 1	104 (76-174)	141 (103-187)	98 (74-206)	69 (52-85)	99 (75-141)
DAY 2	91 (71-167)	132 (81-185)	89 (67-185)	62 (52-73)	103 (74-124)
DAY 3	85 (69-154)	110 (78-197)	82 (65-139)	60 (47-76)	84 (66-140)
DAY 5-6	82 (64-153)	96 (74-181)	77 (63-140)	62 (47-80)	85 (69-157)
**PLASMA IL-6 [nG/L]**					
DAY 1	234 (109-673)	786 (261-3658)	186 (97-310)	67 (58-359)	173 (120-320)
DAY 2	141 (85-363)	169 (108-409)	97 (51-175)	73 (37-161)	324 (161-597)
DAY 3	105 (48-303)	94 (51-401)	74 (22-129)	53 (38-74)	425 (172-1461)
DAY 5-6	63 (27-174)	47 (34-165)	35 (15-96)	30 (13-63)	259 (142-967)
**PLASMA NGAL [µG/L]**					
DAY 1	433 (226-860)	1032 (577-1386)	393 (213-680)	220 (191-228)	257 (174-334)
DAY 2	373 (230-653)	720 (459-1183)	350 (229-488)	152 (103-237)	278 (197-376)
DAY 3	345 (215-569)	631 (352-904)	304 (206-466)	125 (83-208)	302 (225-499)
DAY 5-6	328 (215-526)	395 (291-669)	260 (206-430)	121 (81-192)	320 (197-651)

**PLASMA LACTATE [MMOL/L]**					
DAY 1	2.3 (1.5-3.4)	3.5 (2.4-5.2)	1.7 (1.2-2.5)	2.8 (2.6-2.8)	2.2 (1.6-3.5)
DAY 2	1.7 (1.3-2.2)	2.0 (1.5-2.9)	1.5 (0.9-1.8)	1.0 (0.8-1.3)	2.0 (1.8-3.6)
DAY 3	1.4 (1.0-2.0)	1.6 (1.3-2.2)	1.2 (0.9-1.4)	0.9 (0.9-1.0)	2.0 (1.3-4.8)
DAY 5-6	1.3 (0.9-1.7)	1.4 (1.1-1.8)	1.1 (0.8-1.5)	2.0 (1.7-2.2)	1.3 (1.0-1.5)
**BLOOD PaO_2 [kPA]**					
DAY 1	10.1 (7.5-12.8)	10.1 (9.3-12.7)	9.2 (7.0-11.0)	6.6 (4.2-10.5)	14.0 (10.5-23.9)
DAY 2	9.4 (8.2-11.2)	9.9 (9.2-11.1)	8.5 (6.2-10.2)	9.0 (6.1-10.1)	10.6 (9.1-13.6)
DAY 3	8.9 (7.1-10.9)	9.2 (7.6-10.8)	7.8 (6.3-9.3)	11.4 (8.2-16.3)	11.0 (10.2-12.0)
DAY 5-6	8.8 (6.4-10.7)	9.1 (6.9-11.2)	7.2 (5.7-8.8)	7.7 (6.3-9.1)	11.5 (10.1-13.0)
**BLOOD BASE EXCESS [MMOL/L]**					
DAY 1	-1.8 (-4.8-1.05)	-5.0 (-7.2- -1.4)	-0.8 (-3.4-2.5)	1.4 (0.0-2.7)	-2.2 (-6.0-1.0)
DAY 2	-0.5 (-2.5-2.5)	-1.9 (-3.5- -0.4)	0.6 (-2.0-2.6)	0.5 (0.5-1.5)	1.0 (-2.5-2.6)
DAY 3	1 (-2.175-3.775)	-0.2 (-3.8-2.7)	1.2 (-1.7-4.1)	0.3 (-0.5-0.8)	3.0 (0.6-4.8)
DAY 5-6	3.1 (0.8-6.2)	2.0 (0.5-5.6)	3.8 (1.3-6.1)	1.2 (0.4-1.9)	5.7 (4.0-6.6)
**WBC $X10^9/L$**					
DAY 1	15.8 (11.0-21.5)	16.2 (6.0-21.5)	16.2 (11.1-20.6)	17.1 (11.2-20.6)	13.8 (12.4-29.8)
DAY 2	13.3 (8.5-19.3)	18.6 (13.5-24.3)	11.6 (8.4-17.0)	10.5 (5.2-15.2)	11.8 (6.7-13.5)
DAY 3	10.6 (6.0-18.0)	18.3 (12.2-22.0)	9.6 (7.0-14.3)	7.7 (6.0-9.0)	5.6 (4.4-11.1)
DAY 5-6	9.8 (6.0-14.3)	13.9 (9.9-17.3)	9.0 (6.3-12.7)	8.5 (7.2-9.3)	6.7 (4.9-12.2)

CRP; C-reactive protein, PCT; pro-calcitonin, PLT; platelets, IL; interleukin; NGAL; neutrophil gelatinase associated lipocalin, PaO2; partial pressure of oxygen, WBC; white blood cell count.
